# Supplementary material for: Effect of circle, surface type and stride duration on vertical head and pelvis movement in riding horses with pre-existing movement asymmetries in trot
Source: PLoS One. 2024 Aug 16;19(8):e0308996. doi: 10.1371/journal.pone.0308996 (PMC11329133; doi:10.1371/journal.pone.0308996)
Supplement: Attachment — Submitted filename: PONE-D-23-26830.docx [file pone.0308996.s002.docx]

Effect of circle, surface type and stride duration on vertical head and pelvis movement

in ridden horses with pre-existing movement asymmetries identified during a straight line

trot

This study builds on previous work to explore some of the practical challenges of measuring/analysing the results of vertical head and pelvic movement asymmetries during a clinical examination. Because the work is taken from another study I find the methods lacking and end up with a number of questions related to the research design that need clarification. The use of a soft surface would have been enhanced if the authors had defined ‘soft’ a little better. This distinction may also be a reason for the lack of significance of this factor. The discussion becomes rather long and unclear as it develops and I feel the clinical implications are largely not clinical implications, but further discussion. This section needs considerable revision. Currently the conclusion largely follows previous work. Perhaps the authors could consider how their analysis of data could be improved to pull out more meaningful outcomes.

Abstract

Too many results. Stick to the key results instead of including all of them and then explain what they mean instead of just writing a very general conclusion, which is really just another way of stating the results.

Line 70-71: Increased on a hard surface compared to what?

Line 71: the not this, and remove s

Line 71-73: Is this in a straight line? Please clarify.

Line 73: why potential?

Line 76 to 77: Horses may trot at different speeds or something like that instead of the speed might vary.

Line 79: Also, why is this? How was the difference in speed explained by these authors?

Line 90 to 92: Technically these are factors related to lunging compared to straight line motion asymmetries. No sure that ‘exercise related’ is really the right way to describe your factors.

Line 93: H1. What do you mean by direction and where is this justified as a hypothesis in your introduction?

Line 95: H2. You have already stated this has been found in Lines 51 to 54 from previous studies, so I am not sure how this hypothesis contributes additional knowledge?

Line 117-118: Reference for stride splitting?

Line 121 to 126: Please expand on your sign convention, so it is very clear when asymmetry is increased.

Line 129: Not clear in the introduction why circle diameter was introduced into the design.

Line 131: Define what you mean by fast

Line 136: More information is needed about the ‘soft’ surfaces. How soft was soft?

Line 137 to 140: If there were 16 conditions and the horses were already identified as having asymmetries in a straight line, how did you measure the conditions? How do you know that the increases in asymmetry are not a result of the order in which you collected the data?

Line 142: The inclusion criteria is somewhat confusing. You have said that the horses were in full training and considered sound by their owner. Then you go on to state that any judged as 2 or more out of 5 were excluded and that all had movement asymmetries above the Lameness Locator thresholds. Were the horses still in training on the day they came in for the testing and the owners considered them to be sound when they came in? Or did they come in for a veterinary assessment because they had become asymmetric/lame and were then included in the study if the owner agreed? This is very important. Please revise to clarify.

Line 150: Referring to another study related to the trial days and how each horse was grouped is not appropriate here, as it raises many more questions. Did the movement asymmetry change between days for straight line trot? If so, this suggests that these horses were not consistently asymmetric, which is quite different to a horse being consistently asymmetric (which would suggest sub-clinical or clinical pathologies). Non consistent asymmetry might not provide you with relevant results.

How many days were between trial days?

If horses had more than one predominant asymmetry, were they unilateral or multi-limb lame, as this may affect your results?

Please be very clear about what you did.

Line 175 to 181: Why? I don’t follow your reasoning here (as above).

Line 202: Not sure direction is the best description for inside-outside limbs on a circle/circle size/straight line. I am also wondering why straight is here. I thought you wanted to compare the difference between other conditions and straight?

Line 334-335: Your description of ‘movement direction’ which relates to inside/outside limbs probably needs to state inside/outside limb (where relevant) throughout the discussion for clarity.

Line 398 to 402: This statement (and actually Figure 2) might suggest that in order to unpick changes due to exercise condition related to head movement you may have been better to evaluate the difference between straight line and the other conditions.

Line 406: Inside and outside hindlimbs?

Line 454 – 456: Again, it depends on what the meaning of a ‘soft’ surface is.

Line 476 to 482: It is not clear what you are trying to say here. Please revise.

Line 483-503: It is very unclear what points you are trying to make here. This section is too long and lacks focus.

Line 513 to 529: What has this got to do with clinical implications? The only thing I am taking home from that sat the moment is that you are suggesting that horses with greater movement asymmetries/lameness should be trotted faster when they are assessed. Is this really your take home message? In addition, please be careful when reporting on studies that have found differences (in speed for example) with sound horses at much faster speeds compared to lame horses.

Line 530 to 547: If head asymmetry can change by up to the threshold, then surely that could mean that a horse trotted faster could exceed the threshold compared to the same horse that was trotted slower where a 3 mm difference was found? Would it not be a good idea to look at improving how speed is regulated?

Conclusion is rather long and mainly supports other work without really pulling out new and important information. Consider revising.

.
