# Supplementary material for: Effect of circle, surface type and stride duration on vertical head and pelvis movement in riding horses with pre-existing movement asymmetries in trot
Source: PLoS One. 2024 Aug 16;19(8):e0308996. doi: 10.1371/journal.pone.0308996 (PMC11329133; doi:10.1371/journal.pone.0308996)
Supplement: Attachment — Submitted filename: Response to Reviewers.docx [file pone.0308996.s003.docx]

Effect of circle, surface type and stride duration on vertical head and pelvis movement

in ridden horses with pre-existing movement asymmetries identified during a straight line

trot

**REVIEW 1**

This study builds on previous work to explore some of the practical challenges of measuring/analysing the results of vertical head and pelvic movement asymmetries during a clinical examination. Because the work is taken from another study I find the methods lacking and end up with a number of questions related to the research design that need clarification. The use of a soft surface would have been enhanced if the authors had defined ‘soft’ a little better. This distinction may also be a reason for the lack of significance of this factor. The discussion becomes rather long and unclear as it develops and I feel the clinical implications are largely not clinical implications, but further discussion. This section needs considerable revision. Currently the conclusion largely follows previous work. Perhaps the authors could consider how their analysis of data could be improved to pull out more meaningful outcomes.

*Thank you for the constructive feedback. The manuscript has been substantially amended to provide better rational and more detail with regards to the methodology. The discussion has also been revised and streamlined.*

**ABSTRACT**

Too many results. Stick to the key results instead of including all of them and then explain what they mean instead of just writing a very general conclusion, which is really just another way of stating the results.

*This has now been edited.*

**INTRODUCTION**

*The introduction has been changed substantially to address the points raised below.*

Line 70-71: Increased on a hard surface compared to what?

Line 71: the not this, and remove s

Line 71-73: Is this in a straight line? Please clarify.

Line 73: why potential?

Line 76 to 77: Horses may trot at different speeds or something like that instead of the speed might vary.

Line 79: Also, why is this? How was the difference in speed explained by these authors?

Line 90 to 92: Technically these are factors related to lunging compared to straight line motion asymmetries. No sure that ‘exercise related’ is really the right way to describe your factors.

Line 93: H1. What do you mean by direction and where is this justified as a hypothesis in your introduction?

Line 95: H2. You have already stated this has been found in Lines 51 to 54 from previous studies, so I am not sure how this hypothesis contributes additional knowledge?

**METHODOLOGY**

Line 117-118: Reference for stride splitting?

*This has now been added.*

Line 121 to 126: Please expand on your sign convention, so it is very clear when asymmetry is increased.

*This has now been clarified (lines 124-130)*

Line 129: Not clear in the introduction why circle diameter was introduced into the design.

*This has now been clarified in the introduction.*

Line 131: Define what you mean by fast

*Clarified* (line 214-218)

Line 136: More information is needed about the ‘soft’ surfaces. How soft was soft?

*The ‘soft surface’ was either sand, sand and woodchip or sand and fibre (consistent for each individual horse between straight line and lungeing). This was described in the manuscript (original lines 136-137, now line 220). While we appreciate that that there might be a variation between different types of soft or hard surface, the types of surface and their classification as ‘soft’ or ‘hard’ is representative of what would be using during* *orthopaedic evaluation in the field.*

Line 137 to 140: If there were 16 conditions and the horses were already identified as having asymmetries in a straight line, how did you measure the conditions? How do you know that the increases in asymmetry are not a result of the order in which you collected the data?

*The order was randomized for each horse – this has now been clarified in the manuscript (line 226). Most horses were screened one day and then first data collection started one or a couple of days later. To be included in the original study, a horse had to be asymmetric on straight line hard surface during the slower trot (preferred speed) on the first data collection day (prior to NSAID or placebo treatment). Some horses had their data collection and screening on the same day and for those, the conditions were in random order and then they were included in the study if the straight hard slow condition was above the described thresholds. The horses were then reassessed three more times (as described in the methodology). Consequently, for the present study we had asymmetry measurements from three different dates available for each horse (excluding any post-NSAID measurements) and these were then used to evaluate the predominant asymmetry on each day.*

Line 142: The inclusion criteria is somewhat confusing. You have said that the horses were in full training and considered sound by their owner. Then you go on to state that any judged as 2 or more out of 5 were excluded and that all had movement asymmetries above the Lameness Locator thresholds. Were the horses still in training on the day they came in for the testing and the owners considered them to be sound when they came in? Or did they come in for a veterinary assessment because they had become asymmetric/lame and were then included in the study if the owner agreed? This is very important. Please revise to clarify.

*Additional explanation has been added (line 136 onwards). This was a convenience sample of riding horses considered free from lameness by their owner. However, some of the horses had to be excluded as they were deemed too lame to continue in their normal training by the veterinarians involved in the data collection.*

Line 150: Referring to another study related to the trial days and how each horse was grouped is not appropriate here, as it raises many more questions. Did the movement asymmetry change between days for straight line trot? If so, this suggests that these horses were not consistently asymmetric, which is quite different to a horse being consistently asymmetric (which would suggest sub-clinical or clinical pathologies). Non consistent asymmetry might not provide you with relevant results.

*We wanted to assess how these mild but consistent asymmetries (above Lameness Locator thresholds and with the mean value greater than 1.25 standard deviation) change under different conditions on the same day so variation between days does not impact this. Horses with clinical pathologies can also show variable degree of asymmetry between days. Around 50% of the horses in this study were very consistent in their asymmetries over time while others were not.*

How many days were between trial days?

*Clarification added (lines 152-153). This varied depending on when which measurement was used. This could have been from 4 days apart (if pre-placebo and placebo trials were included – this was the case for 14 horses) or up to 24 days apart.*

If horses had more than one predominant asymmetry, were they unilateral or multi-limb lame, as this may affect your results?

Please be very clear about what you did.

*This is unknown as the horses did not go through a full lameness workup. The study population was a selection of horses that were presumed sound by their owners and were in full work but still presented with movement asymmetries above thresholds that are often used for lameness screening. These thresholds were based on the degree of movement asymmetry comparable to about 70% of horses in a similar population of presumed sound horses in a study by Rhodin et al. (2017). In this study, the aim was to investigate the change in the predominant movement asymmetry parameter on that given day. As mentioned above, around 50% of the horses were consistent with their movement patterns but other horses were not.*

Rhodin M, Egenvall A, Haubro Andersen P, Pfau T. Head and pelvic movement asymmetries at trot in riding horses in training and perceived as free from lameness by the owner. Loor JJ, editor. PLoS ONE. 2017;12: e0176253. doi:[10.1371/journal.pone.0176253](https://doi.org/10.1371/journal.pone.0176253)

Line 175 to 181: Why? I don’t follow your reasoning here (as above).

*Clarification provided in the responses above.*

Line 202: Not sure direction is the best description for inside-outside limbs on a circle/circle size/straight line. I am also wondering why straight is here. I thought you wanted to compare the difference between other conditions and straight?

*This term has been used consistently in previous papers (examples provided below) therefore this is not a novel approach to classification when horses are evaluated during lungeing. However, additional explanations have been added for more clarity throughout the manuscript.*

*To enable a comparison between the lungeing conditions and straight line condition, all the conditions (including straight) had to all be included in the model.*

Pfau, T.; Scott, W.M.; Sternberg Allen, T. Upper Body Movement Symmetry in Reining Quarter Horses during Trot In-Hand, on the Lunge and during Ridden Exercise. Animals 2022, 12, 596. https://doi.org/10.3390/ ani12050596

Pfau, T.; Bolt, D.M.; Fiske-Jackson, A.; Gerdes, C.; Hoenecke, K.; Lynch, L.; Perrier, M.; Smith, R.K.W. Linear Discriminant Analysis for Investigating Differences in Upper Body Movement Symmetry in Horses before/after Diagnostic Analgesia in Relation to Expert Judgement. Animals 2022, 12, 762. https://doi.org/10.3390/ani12060762

**DISCUSSION**

Line 334-335: Your description of ‘movement direction’ which relates to inside/outside limbs probably needs to state inside/outside limb (where relevant) throughout the discussion for clarity.

*Additional clarification/explanation has been added where relevant.*

Line 398 to 402: This statement (and actually Figure 2) might suggest that in order to unpick changes due to exercise condition related to head movement you may have been better to evaluate the difference between straight line and the other conditions.

*This part of the discussion has now been amended. The 3-way interaction is significant in the model but the pattern is unclear and little difference is seen in the estimated marginal means (now included in Table 2). However, as the 3-way interaction is significant, it is not possible to interpret the direction-surface interaction (i.e. how surface a circular motion affect head asymmetries) without considering changes in stride duration which makes unpicking the influence of the individual factors challenging.*

Line 406: Inside and outside hindlimbs?

*This has now been amended for more clarity.*

Line 454 – 456: Again, it depends on what the meaning of a ‘soft’ surface is.

*This is true as there are many types of ‘soft’ surface. Nonetheless, this ‘rough’ classification of types of surface is used in the veterinary textbooks and therefore this is used for general introduction of this paragraph.*

Line 476 to 482: It is not clear what you are trying to say here. Please revise.

*This section has been amended for more clarity.*

Line 483-503: It is very unclear what points you are trying to make here. This section is too long and lacks focus.

*This section has been streamlined and amended for more clarity.*

Line 513 to 529: What has this got to do with clinical implications? The only thing I am taking home from that sat the moment is that you are suggesting that horses with greater movement asymmetries/lameness should be trotted faster when they are assessed. Is this really your take home message? In addition, please be careful when reporting on studies that have found differences (in speed for example) with sound horses at much faster speeds compared to lame horses.

Line 530 to 547: If head asymmetry can change by up to the threshold, then surely that could mean that a horse trotted faster could exceed the threshold compared to the same horse that was trotted slower where a 3 mm difference was found? Would it not be a good idea to look at improving how speed is regulated?

*This section has been streamlined and amendments made to make it more relevant.*

Conclusion is rather long and mainly supports other work without really pulling out new and important information. Consider revising.

*This section has been amended*

**REVIEWER 2**

*Thank you for the constructive feedback and suggestion on how to improve this manuscript. The methodology section has been amended to provide more detail and clarification is included in the comments below.*

**ABSTRACT**
14 could this sentence be made less vague? Aka …different conditions, like circles and various surfaces, yet better understanding of how these alter movement asymmetries is needed.
*This has now been amended.*

17 how did you control changing stride duration? Looking for this later, also were horses asymmetric for the same or different reasons? The underlying cause of the asymmetry is potentially influential

*The horses were first trotted at their preferred speed and then the handler was instructed to trot the horse at a faster speed. Stride duration was recorded for each run and the run with the higher stride duration was classified as ‘slow’ and lower stride duration as ‘fast’. This has now been further clarified in the methodology.*

*The reason for the asymmetries is unknown as the horses did not go through a full lameness workup. The study population was a selection of horses that were presumed sound by their owners and were in full work but still presented with movement asymmetries above thresholds that are often used for lameness screening (this has now been clarified in the methodology). These thresholds were based on the degree of movement asymmetry comparable to about 70% of horses in a similar population of presumed sound horses in a study by Rhodin et al. (2017). In this study, the aim was to investigate the change in the predominant movement asymmetry parameter on that given day.*

Rhodin M, Egenvall A, Haubro Andersen P, Pfau T. Head and pelvic movement asymmetries at trot in riding horses in training and perceived as free from lameness by the owner. Loor JJ, editor. PLoS ONE. 2017;12: e0176253. doi:[10.1371/journal.pone.0176253](https://doi.org/10.1371/journal.pone.0176253)

18 What discipline?

*Additional description of the population now included in results. For brevity, detailed description of the population is not included in the abstract but a line has been added to say that these are horses in ridden work.*

19 determined via subjective or objective measures? A grade 1 is very inconsistent so how can we know it is consistent enough to be present reliably under the new conditions?

*Wording has been amended for clarity.* *In this study, the aim was to investigate the change in the predominant movement asymmetry parameter on that given day – these were all horses presenting with some movement asymmetries but in full training (see further explanation below).*

24 what was the range? 6 mm to what? 3 mm to what? what did you classify as a grade 1?

*The mean and standard deviations are included for each of the groups in Table 1, range was also added but, for brevity, these are not included in the abstract. The horses were included if they were presenting with movement asymmetries corresponding to subtle to mild lameness (visual scoring). The grading used was an ordinal 0-5 scale ranging from non-lame to non-weightbearing lameness. It is the most commonly used scale in the clinical setting in Sweden. Similar ordinal scale has been used in previous studies, for example Rhodin et al. (2018). In this study, the subjective grading was used to exclude any horses for which it would be unethical to consent for keeping them in training for the following 4 weeks (which was the requirement for the previous study).*

Rhodin M, Persson-Sjodin E, Egenvall A, Serra Bragança FM, Pfau T, Roepstorff L, et al. Vertical movement symmetry of the withers in horses with induced forelimb and hindlimb lameness at trot. Equine Vet J. 2018;50: 818–824. doi:[10.1111/evj.12844](https://doi.org/10.1111/evj.12844)

25 to right front or hind limb? Or ?

*This has now been amended for clarity.*

**INTRODUCTION**
69-71 confusing repetitive sentence and not sure what the s is at the end? Size?

*Now amended.*

121 why didn’t you use the Q score for the head?

*We appreciate that the Q score, which combines the HDmin and HDmax parameters, is often used in the clinical practice. In our experience, the HDmax is a complex parameter that often goes together with HDmin in a classical weight bearing forelimb lameness but in some horses who have a clearly dominant HDmax, we are much more uncertain of how to interpret the parameter clinically. The aim of this study was to evaluate how the different types of movement asymmetries which were present in horses in training change under different conditions, therefore, a combined Q score would not capture this.*

129 how did you ensure the diameter ?

*Markers were placed on the lunge line. This has now been clarified in the methodology (line 213).*

131 what were the cut offs to distinguish between slow and fast? A 10 percent difference? More?

*No minimum difference was required. The horses were first trotted at their preferred speed and then the handler was instructed to trot the horse at a faster speed. Stride duration was recorded for each run and the run with the higher stride duration was classified as ‘slow’ and lower stride duration as ‘fast’.*

136 how many on dirst vs aphalt?

*We did not run an analysis for different types of ‘hard’ surface therefore dirt and asphalt were entered as ‘hard’ surface. For all horses, the surface was consistent throughout the data collection, i.e. they were evaluated on the same type of surface both during a straight line trot and lungeing. While we appreciate that that there might be a variation between different types of soft or hard surface, the types of surface and their classification as ‘soft’ or ‘hard’ is representative of what would be using during* *orthopaedic evaluation in the field.*

144 is this the aaep scale? Reference?

*No, it is not the AAEP scale; it is an ordinal 0-5 scale ranging from non-lame to non-weightbearing lameness. It is the most commonly used scale in the clinical setting in Sweden). Similar ordinal scale has been used in previous studies, for example Rhodin et al. (2018).*

Rhodin M, Persson-Sjodin E, Egenvall A, Serra Bragança FM, Pfau T, Roepstorff L, et al. Vertical movement symmetry of the withers in horses with induced forelimb and hindlimb lameness at trot. Equine Vet J. 2018;50: 818–824. doi:[10.1111/evj.12844](https://doi.org/10.1111/evj.12844)

146 unclear what at the discretion of the study vets meant? Were they or weren’t they allowed?

*This has now been clarified in the manuscript. The horses were excluded if they were considered too lame to continue in their ridden work subject to the judgment of the veterinarians conducting the study.*

147 how far out from an NSAID were they?

*This section has now been amended with more detail provided on the washout period (14-16 days) – line 152-153.*

164-170 this font seems smaller?

*This is the figure legend. Formatting amended so that the text follows the Fig Title.*

164 I thought they weren’t looked at while actively getting the NSAID?

167 I think you can leave the NSAID time point out and just discuss 3 times, else it is confusing

*This is a figure legend to explain the previous study design and inclusion criteria for the present study.*

167-170 is not a complete sentence

*This is a figure legend. Formatting amended so that the text follows the Fig Title.*

104 need a section on where the horses came from? State the N even if in the other paper need some basic details here. I’m expecting breed, discipline, and more on their lameness in the results

*Additional detail about how horses were recruited has been added (line 138 onwards). These were horses in full work considered sound by their owners. These horses did not undergo a full clinical lameness exam but grading was used so that horses considered too lame to continue their normal work (>2 degrees lame assess visually) were excluded – these horses were advised to undergo lameness assessment and were not included in the study.*

*Summary of breed, age, discipline, etc. is now included in the results section.*

189 unclear what inverted means? You used it twice vs explaining it , are we talking side? Or positive or negative? Or a fraction?
*This has now been clarified (lines 200-202).*

**RESULTS**
Need descriptors for the animals, some indication of discipline, site of lameness may cause them to behave in different ways, signalment, breed etc.

*Age, breed and discipline have now been added for better description of the population. These horses did not undergo full clinical lameness exam and therefore the source of the asymmetries is unknown. Additional clarification in response to line 17 above.*

Did you assess for normality?

*Yes, histograms of residuals were inspected for normality – this was previously mentioned in the methodology but a comment has now been added to confirm normal distribution was confirmed.*

Table 1 put the data regarding slow st line etc above the table

*Now updated*.

PD min seems to go below 3 when you look at the SD?

*This is correct. Because we selected for horses above the 3mm threshold, there is a greater number of horses that are above the mean value of 6.4mm therefore resulting in SD of 3.7. Range is now included in the table to provide more detail about the spread of the asymmetry values.*

234 was stride duration really the same front and hind ?

*Strictly speaking only the right forelimb stride duration was measured by the gyroscope attached to the dorsal aspect of P1 of that limb. While stance duration might differ slightly stride duration is not expected to.*

Did you see statistically sig dif in stride duration bweteen conditions? P balues?

*While this was not included in the manuscript, additional mixed models were evaluated with stride duration as the outcome variable. Overall, the horses trotted with lower stride duration (faster trot) during a straight line trot compared to lungeing. The stride duration tended to be higher on soft surface but the confidence intervals were overlapping suggesting smaller influence of surface type on stride duration. The means value per condition are included in the manuscript to give the reader an idea about the range of values achieved per condition.*

244 not clear what direction means? Inside or outside as you removed right and left?

244 to 252 can you add in some comment about how it affected things? Like what you did in lines

259-262?

*For the three comments above - the ‘overall effect’ subsection has now been removed the results are now immediate discussed individually for the forelimb and hindlimb related asymmetries. The ‘direction’ term, explanation has been included to clarify and additional explanation given in the results which hopefully provides more clarity.*

Please add N to your tables

*This has been rectified.*

278-282 this sentence is unclear. What is smallest influence mean? Was it significant still ?

*The 3-way interaction is statistically significant but the biological relevance is questionable due to small coefficient estimates and no clear pattern. The results have now been edited to highlight this and confidence intervals added to the estimates in Table 2*.

267 assuming the journal wants a smaller font for the fig?

*This is not specified in the formatting guidelines but we will check with the publisher.*

278 see above re smallest influence etc.

296 I think the in interaction needs to be placed elsewuere to make more sense? Influenced by the interaction of direction and stide duration.
*This section has now been edited for more clarity.*

Please confirm all trotted and there were no pacers.

*Additional information about horses has now been added. No pacers were included.*

**CONCLUSION**

560 I think the term movement direction is unclear and misleading if it is truly straight line vs circling. Can you find a better indicator for it?

*This has now been reworded for better clarity.*

Did you run a power analysis to see how many animals you needed before hand?

*The data used for this study was collected for another study and a power calculation for the outcomes of this study was therefore not performed*

For the discussion there are no limitations described. please add

*Limitations section has been added.*

Some commentary should be addressed to the cause of the asymmetry and what impact that can have on the parameters. WHat if there is bilateral hind limb lameness - what might that look like while circling etc.

*This has now been added to the new ‘limitations’ section.*
