# Supplementary material for: Effect of circle, surface type and stride duration on vertical head and pelvis movement in riding horses with pre-existing movement asymmetries in trot
Source: PLoS One. 2024 Aug 16;19(8):e0308996. doi: 10.1371/journal.pone.0308996 (PMC11329133; doi:10.1371/journal.pone.0308996)
Supplement: Attachment — Submitted filename: Effect of circle.docx [file pone.0308996.s004.docx]

Effect of circle, surface type and stride duration on vertical head and pelvis movement

3 I’d leave off the identified during st line at the trot in the title- this is confusing as you say effect of circle…. Then straight line trot. I’ve fine with just pre-existing movement asymmetries.

14 unclear what upper body movement asymmetries is? This makes it sound like you mean a lameness of the shoulder, hip or pelvis. I’d remove upper body minimally and work to clarify this

18 I’d make mention of pre-existing asymmetries here somehow. After pelvis would seem appropriate

20 unclear what predominant asymmetries is? Do you mean front vs hind limb or objective degree of asymmetry? Aka the more lame together and the less lame/asymmetric together?

23 surfaces

24 for later, how was slow and fast set? How were these compared between horses in real time?

40 do we have diagnoses for these horses? Aka were all OA or all soft tissue? Before making a claim that surface doesn’t matter we would need to know this, as this statement can be misleading without that knowledge

48 looks like an extra space

65 a hard surface

66 a soft surface

71-72 how is your study different then if this has been established?

77 any pd min results?

I’d clarify if these asymmetries are considered clinically relevant by veterinarians even if the clients didn’t think so.

123 did you look at the q score?

162 did predominant asymmetries change between days? If so more details are needed.

Esp did asymmetries change if on the circle from the main one seen at the straight line?
