# Supplementary material for: Effect of circle, surface type and stride duration on vertical head and pelvis movement in riding horses with pre-existing movement asymmetries in trot
Source: PLoS One. 2024 Aug 16;19(8):e0308996. doi: 10.1371/journal.pone.0308996 (PMC11329133; doi:10.1371/journal.pone.0308996)
Supplement: Attachment — Submitted filename: Reviewer comments R2 Final.docx [file pone.0308996.s005.docx]

**Reviewer #2: Effect of circle, surface type and stride duration on vertical head and pelvis movement**

3 I’d leave off the identified during st line at the trot in the title- this is confusing as you say effect of circle…. Then straight line trot. I’ve fine with just pre-existing movement asymmetries.

*Response: The title has now been edited.*

14 unclear what upper body movement asymmetries is? This makes it sound like you mean a lameness of the shoulder, hip or pelvis. I’d remove upper body minimally and work to clarify this

*Response: Now edited and replace with ‘head and pelvis vertical movement asymmetries’ for clarity.*

18 I’d make mention of pre-existing asymmetries here somehow. After pelvis would seem appropriate
20 unclear what predominant asymmetries is? Do you mean front vs hind limb or objective degree of asymmetry? Aka the more lame together and the less lame/asymmetric together?

*Response: The abstract has now been amended to add clarity.*

23 surfaces

*Response: Edited*

24 for later, how was slow and fast set? How were these compared between horses in real time?

*Response: For brevity, in the abstract we only mention that his was determined by stride duration. This is explained in more detail in the methods section. There was no minimum difference in stride duration required – during data collection the aim was to collect two trials with different speeds, the ‘slow’ trial was the one with higher stride duration and ‘fast’ trial was the one with lower stride duration. The slow and fast is therefore assigned within horse not as above/below a certain absolute value across a dataset. In the model, we account for this as we use ‘horse’ as a random factor to account for the variation between horses.*

40 do we have diagnoses for these horses? Aka were all OA or all soft tissue? Before making a claim that surface doesn’t matter we would need to know this, as this statement can be misleading without that knowledge

*Response: As specified earlier in the abstract, these were horses in ridden work, considered sound by their owners but with movement asymmetries above the objective system threshold, as it the case for many horses in normal working population of horses. Consequently, full lameness examination was not conducted within the scope of the study. Population reference (‘horses in full ridden work’) has now been added to this sentence. However, we appreciate that the results could be different for horses presenting with certain pathologies - an additional sentence has been added to highlight that further studies should be carried out in clinically lame horses on this topic.*

INTRO:

48 looks like an extra space

65 a hard surface

66 a soft surface

*Response: All now corrected.*

71-72 how is your study different then if this has been established?

*Response: The introduction has now been edited to better highlight where the current gaps in research are. No previous studies evaluated horses that presented with movement asymmetries on different types of surface and/or varied the circle size. Since surface type and circle diameter often vary between multiple evaluations of the same horse (due to inconsistent handling) or between different locations, it is useful to know how much these factors influence the movement asymmetries.*

77 any pd min results? I’d clarify if these asymmetries are considered clinically relevant by veterinarians even if the clients didn’t think so.

*Response: This section has now been made more concise to these results are no longer discussed in detail.*

123 did you look at the q score?

*Response: We appreciate that the Q score, which combines the HDmin and HDmax parameters, is often used in the clinical practice. In our experience, the HDmax is a complex parameter that often goes together with HDmin in a classical weightbearing forelimb lameness but in some horses who have a clearly dominant HDmax, we are much more uncertain of how to interpret the parameter clinically. The aim of this study was to evaluate how the different types of movement asymmetries which were present in horses in training change under different conditions, therefore, a combined Q score would not capture this.*

162 did predominant asymmetries change between days? If so more details are needed. How many changed from front to hind etc if not reported below
Esp did asymmetries change if on the circle from the main one seen at the straight line?

*Response: This was not within the scope of this study. This study investigates how different circle size, speed and surfaces impact the movement asymmetries within the same day. For each horse we only use data from the measurements taken on the same day as the baseline which was used for assigning the predominant asymmetry. As some horses were consistent and some switched, it would be interesting explore whether there were differences between the horses with consistent and inconsistent predominant asymmetry between the days. However, this would have resulted in much smaller number of horses per predominant asymmetry group. In the future, this could be explored in a bigger dataset.*

214- what was the magnitude of the difference between the two? How did that range between horses? Was any horse fast as a default? How did they slow down with time?

*Response: No minimum difference was required. The horses were first trotted at their preferred speed and then the handler was instructed to trot the horse at a faster speed (under the same ‘surface’ and ‘direction’ conditions). This was then verified objectively via the stride duration. For the analysis, we use mixed models with horse as a random factor to account for the individual variation so the differences between horse would not affect the results. The exercise conditions were randomized to minimise the effect of order.*

*For completeness, difference between the minimum and maximum stride duration recorded within the individual horses is included in the results section. Within the individual horses, the stride duration changed between 68 ms and 227 ms across all the condition (mean for all the horses was 128 ms). This means some horses changed their stride duration only slightly (68 ms) regardless of whether they were trotted in hand or lunged or whether the trotted on different types of surface horses but some horses had a much bigger range (up to 227 ms). In a clinical setting this range would be much smaller as horses are usually evaluated at their preferred trotting speed while in this study they were purposefully asked to trot faster the second time round.*

219 how did you maintain either a 10 or 15 m circle assuredly?

*Response: Markers were placed on the lunge line to ensure consistency. This is mentioned in the methodology (previously line 213, now line 138 as this section has been reorganised)*.

221 how different were the surface types and how many different types were there (aka how many barns were evals performed at? Were evals at the same barn performed in the same spot)? Surface differences should be mentioned in limitations.

*Response: If the horses were from the same establishment, they were all evaluated under the same conditions. There horses came from 10+ locations with varying types of surface. The exact specification of ‘soft’ and ‘hard’ surface varied between locations (lines 141-143) but the surface was kept consistent for each horse, i.e. they were evaluated on the same soft or hard surface across the conditions (for both straight line and lungeing). While we appreciate that that there might be a variation between different types of soft or hard surface, the types of surface and their classification as ‘soft’ or ‘hard’ is representative of what would be used during* *orthopaedic evaluation in the field. And additional paragraph has been added to limitations section to address this point.*

RESULTS:
245 do you have BCS scores? Breeds? Lameness eval diagnoses?

*Response: BCS was not recorded. Breed information has now been added. As for the lameness evaluation, these were horses considered sound and in normal work that were measured for another project evaluating the effect of NSAID on horses in ridden work with movement asymmetries above commonly used clinical thresholds. A full lameness investigation was not performed within the scope of the original study.*

268 I’d re mention what stride duration is a proxy for here

*Response: Edited.*

DISCUSSION
362-363 without knowledge of the cause of the assymetries I don’t feel we can make this conclusion as broad sweeping as it is.

*Response: References to the specific population used in this study added throughout the discussion.*

366 a summary sentence with speed should be made here aka the faster they go the more asymmetry or whatever the finding is.

*Response: Edited.*

383 here again, the LLis calling them lame, but are they clinically lame? This needs to be clarified. The owners thinking they are not lame is not sufficient.

*Response: We only now that they are asymmetric above the thresholds proposed for clinical practice by the system used in this study. As full lameness investigation was not performed so it is not possible to say whether these asymmetries are caused by pain/pathology. However, many horses display asymmetries above these thresholds and it does not necessarily mean that they have underlying issues as some of these asymmetries can be due to normal biological variation.*

402 more comparison to previous papers should be undertaken to compare outcome variables to your study

*Response: The discussion has now been significantly amended and the point above has been addressed.*

435-440 please address if these horses are clinically lame as determined by a veterinarian and if so if diagnoses are known. If you aren’t considering them clinically lame how was this determined? How can you say they aren’t clinically lame when objective data is beyond the norm? did they have a full lameness work up?

*Response: The study population was a selection of horses that were presumed sound by their owners and were in full work but still presented with movement asymmetries above thresholds that are often used for lameness screening. Asymmetries above this threshold do not always indicate lameness as asymmetries can also arise due to normal biological variation. Previous studies have shown that there is a no clear cut ‘threshold’ between lame and non-lame horses as there is always an overlap between objective asymmetry values when horses are judged as non-lame horses and lame by clinicians. It is possible that some horses might have had pathologies that were not known but as the horses did not go through a full lameness workup, we cannot confirm this (additional points have been added to the discussion now).*

*In this study, the subjective grading was only used to exclude any horses for which it would be unethical to consent for keeping them in training for the following 4 weeks (which was the requirement for the study for which the data were originally collected). The grading used was an ordinal 0-5 scale ranging from non-lame to non-weightbearing lameness and any horses graded as 3 or more were excluded. This grading is the most commonly used scale in the clinical setting in Sweden. Similar ordinal scale has been used in previous studies, for example Rhodin et al. (2018).*

Rhodin M, Egenvall A, Haubro Andersen P, Pfau T. Head and pelvic movement asymmetries at trot in riding horses in training and perceived as free from lameness by the owner. Loor JJ, editor. PLoS ONE. 2017;12: e0176253. doi:[10.1371/journal.pone.0176253](https://doi.org/10.1371/journal.pone.0176253)

Rhodin M, Persson-Sjodin E, Egenvall A, Serra Bragança FM, Pfau T, Roepstorff L, et al. Vertical movement symmetry of the withers in horses with induced forelimb and hindlimb lameness at trot. Equine Vet J. 2018;50: 818–824. doi:[10.1111/evj.12844](https://doi.org/10.1111/evj.12844)

500 why do you think your results differed?

*Response: The discussion has now been significantly amended and the point above has been addressed.*

540 given all of this, since horses did not have a full lameness eval, statements regarding circle size etc should be always qualified. Why was a full lameness not performed? Were subjective scores assigned at all? Was there a clinical corroboration with the LL? I have seen the LL fail especially with how it uses the law of sides (aka saying it’s a hind limb lameness when the forelimb issue is clearly a real cause of pain when issues present on the same side).

*Response: In the original study, horses were excluded if they were scored as graded 3 or above (out of 5) by clinicians involved in the data collection. This was due to ethical reasons as the horses would have to continue in their regular work if there were to be included in the study. The AIDE results was not used for this. The Lameness Locator was used to quantify head and pelvis movement asymmetries as the horses had to show movement asymmetries about the suggested thresholds to be enrolled in the study. We have chosen these thresholds for consistencies as many previous studies also use the same thresholds when describing horses as symmetrical (below thresholds) or asymmetrical (above thresholds).*

547 how do you recommend this is kept the same? Use stride duration on objective LL? How far beyond the same can it be and still be ok, please discuss this further in the discussion and add in more in results as needed.

548 decreased

*Response: Edited*

553 surface type needs qualification aka unknown primary cause of asymmetry

*Response: Quantification of population now added.*

Reviewer #3: This study aimed to determine the effect of several conditions, relating to surface type, trot stride duration, lunging diameter, and movement type (straight line vs. circular motion), on upper-body movement asymmetry parameters. The study is interesting and relevant, particularly as it has the potential to inform clinical practice. It is obvious that the authors have made significant changes to this manuscript, which should be applauded. However, the manuscript is still far too long and repetitive, making it very difficult to follow and thus to draw out the most important/relevant information. Further, clarity and conciseness are an essential element for any study, but particularly for a study design like this where over 15 different conditions are presented. Unfortunately, this is often not the case with this study, which increases the difficulty in reading, following, and drawing conclusions from such a long and repetitive piece of writing. There are several grammatical errors (particularly comma use) and run-on sentences throughout, which must be addressed. The methods section left me with several unanswered questions and there are several methodological limitations of this study which I do not feel have been adequately addressed/highlighted to the reader. I have attempted to highlight my more specific concerns in the points below.

INTRODUCTION:

*Response: Thank you for the suggestions below. Significant portion of the introduction has now been edited to address the point below.*

Line 50: do you mean to say that these movement asymmetries are the result of natural/physiological adaptations that occur when a horse moves on a circular path? If so, what might some of these asymmetries on the lunge be caused by (i.e. body angle lean?)? You get into this later, in line 60, but the paragraph could be rearranged for clarity.

Line 55: “inside” – please check grammar and spelling throughout.

*Response: Edited.*

Line 57: not consistent in what sense? Please concisely add more context for the reader here. Not consistent within or between studies?

Line 63: “so it is important”

Line 65: secondly to what? Evaluated for what? Hard surface is also often used (albeit less frequently) for lunging, please edit this sentence for clarity.

Line 68: “uneven weightbearing” is this the most accurate description for the source of HDMin asymmetry?

Line 71: “and a small increase” were these increases significant? Same for the next lines?

Line 76: what is meant by “greater changes”? Be specific so that the reader obtains the appropriate level of context/background knowledge from the introduction section.

Line 78: new paragraph.

Line 84: “multiple runs” – define what is meant by this. Some readers may not be familiar with some of the terms used in this field.

Line 86: run on sentence – please revise for clarity and check grammar.
Line 91: “influence”. Also another run on sentence.

Line 93: lunging and circular motion are used interchangeably, which is fine, but probably confusing for the non-familiar reader. It would be useful to describe lunging as an exercise where the horse moves on a circular path around a handler at the beginning of the introduction. Why is this an important part of the lameness work up? This is also relevant information to (concisely) include. In line 93, do you mean that circle diameter will have a greater influence on movement asymmetry than surface …. Or do you mean lunging vs. straight line motion? Clarity needed for the hypotheses.

Line 97: consider using increase instead of amplified.

M&M:
This section lacks flow and is very difficult to follow (for example putting data processing before any description of the included horses and data collection procedures). One simple way to address this is by re-organising the sections (and their content) for clarity. I would suggest re-arranging this section so that the subsections are in the following order: Inclusion Criteria and Data Collection (including protocols for number of trials/runs etc., and a brief description of the proprietary software and asymmetry outputs that are used to define the inclusion criteria) ◊ Data Processing ◊ Data Analysis ◊ Statistical Analysis. Note that the “Allocation to predominant movement asymmetry groups” and “Data normalisation” sections should go under Data Analysis and/or Processing sections.

*Response: Thank you for the suggestions. This section has now been rearranged for better flow and clarity.*

Line 140: were screened.

Line 146: please provide a reference for the scale. This sentence should be revised to state that qualified veterinarians clinically evaluated each horse and those that were subjectively deemed to have a lameness grade >2/5 were excluded from the study. Assuming this is true and that veterinarians assessed each horse. Include the initials of the veterinarians if they are also authors on this manuscript.

*Response: Reference for scale added and a sentence added that full lameness examination was not conducted. There were multiple veterinarians who evaluated the horses in the original study but only one of them is the co-author for this study.*

Line 150 – thresholds.

*Response: Edited.*

Line 148 – 152 – run on sentence. Difficult to follow, particularly for readers unfamiliar with these thresholds/methods. Please revise for clarity.

*Response: This has now been edited.*

I would argue that line 152 – 157 is not necessary, as it does not relate to the data used in this study. Line 157 can be moved to the beginning of this section (before Briefly, in line 138) and the rest removed. You can refer to Figure 1 in this section too.

*Response: The methodology section has now been rearranged and edited for clarity.*

Line 159: data were (data = plural).

*Response: Edited.*

Line 161 – 165: confusing and requires clarity. The irrelevant information about the larger study can be removed for clarity. In lines 159 – 163 it almost reads like all 4 time pointes were used in this study, as the 4-day washout period ensured that any influence of NSAID treatment on movement asymmetry was eliminated. Then you go on to say that actually only 3 of these timepoints were used for this study, so the above lines seem totally irrelevant/confusing (especially as we have Figure 1 and the reference for the larger study to refer to if needed). Remove Lines 159 – 163 and simply state that data from three trial days (PP, P, P-NSAID) were included for screening for predominant movement asymmetries, as this ensured that any treatment effect of NSAIDS were eliminated from this study. What do you mean by “up to three trial days” in line 163: does this mean that not all horses underwent screening over 3 days?

*Response: This has now been edited for clarity. Due to the design of the previous study, data from up to three different days was available for each horse (excluding the day with active NSAID treatment). If they presented with the same type of predominant asymmetry across the different days, they were included only once (in one group). However, if the horse presented with for example PDmin on pre-placebo day but with HDmin on pre-NSAID, they horse would be include in two groups but with data obtained from different days. This is explained in the ‘allocation to predominant asymmetry groups’ subsection.*

Did horses undergo screening for “predominant movement asymmetry” on the same day as data collection for this study? This needs clarification, as the actual procedures for data collection for the current study are very unclear. If data were not collected on the same day then how long between screening and data collection? The authors mention that nearly half of the horses included in this study did not consistently exhibit the same movement asymmetry across screening days, so how might this have influenced data grouping/analysis/interpretation if data were not collected on the same day (i.e. how could you be sure that this horse wouldn’t exhibit a different predominant movement asymmetry on the actual day of data collection)? More context needed here and this does need to be discussed as a limitation of your study if data were not collected on the same day as screening, particularly for horse without a consistent predominant asymmetry.

*Response: All the horses include in this study had head or pelvis movement asymmetries above the Lameness Locator threshold for at least one asymmetry parameter. The predominant movement asymmetry was selected based on the asymmetry values from the straight line, hard surface, slow speed measurement (as baseline). The rest of the measurements included in the model from that horse were from the same day, measured within a time span of 0-45min from that baseline. As mentioned in the manuscript, some of the horses were included in multiple predominant asymmetry groups but these were data from different days, i.e. the data included in the relevant model were only from the day when that horse was displaying the relevant movement asymmetry at baseline condition. If on pre-placebo day, the horse was showing predominant HDmin movement asymmetry, the data from that day would be included in HDmin model. If the same horse was showing PDmin movement asymmetry at baseline on pre-NSAID day, the data from that day would be included in PDmin model. The methodology section has now been edited to provide more clarity.*

Time points and trial day are used interchangeably – be consistent. Are these referring to the same thing? What is meant by trial mean? How many straight line “runs/trials” were conducted per horse? Was this standardised and was the distance/surface/handler standardised across horses for screening?

*Response: This has now been edited for clarity.*

Line 166: “greatest asymmetry” could the authors be certain that this was in fact related to a primary asymmetry or could this have been compensatory movement that resulted in greater asymmetry (i.e. primary hindlimb asymmetry exhibiting a greater compensatory asymmetry in head vertical displacement). If not, how may this have influenced some of your findings once the horse was evaluated across conditions? Is a compensatory asymmetry more likely to change across conditions than the primary source of asymmetry? If so, are your results valid? I realise that this is a difficult question to answer and that the answer is likely reliant on a full lameness evaluation or using induced lameness models, but this is definitely something that should be discussed in your limitations/discussion section.

*Response: The mean compensatory head movement asymmetry is around 0.8-0.9 mm for each mm of primary hindlimb lameness in horses with induced lameness (Rhodin et al., 2013). In addition, the reduction in head asymmetries after hindlimb diagnostic analgesia was smaller relative the reduction in the pelvic asymmetries (Marunova et al., 2022; Phutthachalee et al., 2020). Therefore, it is not common to have much greater compensatory head movement asymmetry than the primary hindlimb related asymmetry. However, some horses might exhibit larger compensatory movement so we cannot rule this out completely as full lameness evaluation was not carried out.*

Rhodin, M., Pfau, T., Roepstorff, L. and Egenvall, A. (2013) ‘Effect of lungeing on head and pelvic movement asymmetry in horses with induced lameness’, *The Veterinary Journal*, 198, pp. e39–e45. Available at: <https://doi.org/10.1016/j.tvjl.2013.09.031>.

Phutthachalee, S., Mählmann, K., Seesupa, S. and Lischer, C. (2020) ‘Upper body movement analysis of multiple limb asymmetry in 367 clinically lame horses’, *Equine Veterinary Journal*, p. evj.13367. Available at: <https://doi.org/10.1111/evj.13367>.

Marunova, E., Hoenecke, K., Fiske-Jackson, A., Smith, R.K.W., Bolt, D.M., Perrier, M., Gerdes, C., Hernlund, E., Rhodin, M. and Pfau, T. (2022) ‘Changes in Head, Withers, and Pelvis Movement Asymmetry in Lame Horses as a Function of Diagnostic Anesthesia Outcome, Surface and Direction’, *Journal of Equine Veterinary Science*, 118, p. 104136. Available at: <https://doi.org/10.1016/j.jevs.2022.104136>.

Line 174: “data were”

*Response: Edited.*

Line 179: HDMin “mean difference across run/trail between the two halves of a stride for vertical displacement…..”? revise this definition, it us cumbersome and confusing.

*Response: This has now been edited (now under ‘Movement asymmetry parameter’s section).*

Line 187: So did you exclude useful data from horses that showed consistent predominant movement asymmetries? Surely these data are key to truly understanding the effect of the different conditions studied here on horses that present with a consistent movement asymmetries, but yet you underrepresent these horses? These additional data seem invaluable and suggest that your dataset is saturated by data from horses with inconsistent movement patters/asymmetry, which increases the complexity of understanding the effects of these different conditions. Please explain. I may be confused, as I’m still unsure as to whether these IMU data were actually included in your analysis or were just used for grouping horses. This requires clarification.

*Response: For each horse we only use data from the measurements taken on the same day as the baseline which was used for assigning the predominant asymmetry. Indeed, some horses were consistent with the asymmetry pattern and some switched and it would be interesting to explore whether there were differences between the horses with consistent and inconsistent predominant asymmetry between the days. However, this would have resulted in much smaller number of horses per predominant asymmetry group. In the future, this could be explored in a bigger dataset or in clinically lame horses.*

*The objective movement asymmetry data were recorded for each exercise condition and used in the model as outcome variable with surface and direction as fixed factors and stride duration as a covariate. Therefore, answering the question of how the asymmetries that we labelled as predominant change due to surface or lungeing with the limb on the inside or outside of a circle. The methodology has been revised to improve clarity.*

Line 210: this information needs to come sooner in the methods section (see my suggestions above). Again – are these data the same as the “screening data”? Were they collected at the same time/day? If not, this information needs to be included in the article.

*Response: This has now been revised.*

213: markers placed on the lunge to denote where the handler should hold the line? How were the handlers instructed to trot the horses faster? What was the subjective indicator of this so that it was kept consistent across horses?

*Response: The sentence about markers on the lunge line has been edited for more clarity (now line 140). The handlers were told to ask the horse to move subjectively faster than the preferred speed that was collected first for each exercise condition. The data was then retrospectively marked as slow and fast based on stride duration. There was no minimum difference in stride duration required – the ‘slow’ trial was the one with higher stride duration and ‘fast’ trial was the one with lower stride duration. No attempt was done to ensure similar speed between horses; the ‘slow’ and ‘fast’ was assigned within horse not as above or below a certain absolute value across a dataset. In the model, we use ‘horse’ as a random factor to account for the variation between horses.*

Line 232 – these conditions should be defined and this should be done earlier in the methods section. For example: the direction condition is inconsistently defined. In line 212 and 227 you refer to left and right rein, but here it is inside/outside. Please clarify/be consistent throughout.

*Response: The methodology section has now been rearranged and edited for clarity.*

RESULTS
Line 250 – “one or more”. As stated above, nearly half of the horses showed inconsistent movement asymmetries. It would be interesting to know the proportion within of each Group of horses with consistent asymmetries and horses that were also included in one or more other groups. Were there patterns here? i.e., did some groups/asymmetry parameters have more horses with consistent asymmetry and did other groups have a greater proportion of horses included in 1 or more other groups? How might this have influenced your findings?

*Response: As per the earlier comment, we were interested in how the predominant asymmetries change on any given day hence this is the approach which was chose. Variation in movement asymmetries over times, either within day or between days, is common (for example Hardeman et al., 2019). While this additional analysis could have been interesting, it was beyond the scope of this study.*

Hardeman, A.M., Serra Bragança, F.M., Swagemakers, J.H., Weeren, P.R. van and Roepstorff, L. (2019) ‘Variation in gait parameters used for objective lameness assessment in sound horses at the trot on the straight line and the lunge’, *Equine Veterinary Journal*, 51(6), pp. 831–839. Available at: <https://doi.org/10.1111/evj.13075>.

Line 255: the text should not repeat information provided in tables or figures, so line 255 – 257 can be removed.

*Response: Edited.*

Lines 264 – 265 also repeat information provided in the text – is this relevant information for a table footer? Please review this throughout as the results section is currently too long.

*Response: Edited.*

Mean ± SD stride duration data should be placed in a table and referred to in the text. By putting these values in a text, the authors can remove lines 267 – 274 and keep only lines 274 – 278 (referring to the table). This will help to shorten this already too lengthy section.

*Response: This has now been addressed and table added.*

Throughout – there should be a space between values and units (i.e. 714 ms). Consider presenting stride duration in s, which is arguably the norm. Descriptive stats should be presented as 714 ± 46 ms (example from line 268).

*Response: The reporting standards for means and standard deviation vary. We have now double checked the guidelines for PLOS One and they follow SAMPL which recommends using ‘mean (SD)’ annotation.* *The units formatting has now been checked and amended throughout. As for the use ms instead of s, while s is an SI unit, many previous papers use ms instead as changes in stride duration are less than 1s and using ms instead might be more meaningful in this case.*

Line 278: mean range? What do you mean by this. Do you mean mean stride duration of 128 ms (range: 68 – 227 ms). Please check formatting for correctness throughout. Did you run any statistics on stride duration data between conditions? Surely this is important information. Was stride duration affected by circle diameter? It seems like there is some picking and choosing of what conditions are presented and what conditions are not in results. This should be addressed.

*Response: Line 278 reworded for more clarity. These values are provided to give the reader and indications of how much the stride duration could vary within the individual horses across all the conditions (the difference between minimum and maximum stride duration across all conditions). These values are later used in a discussion to give realistic examples the expected change in movement asymmetries due to stride duration variation.*

*The analysis in this manuscript is focused on the effect on the movement asymmetries from different factors. We present output for all significant factors in the models with stride duration included as a covariate. No statistical tests were carried out to compare stride duration between the exercise condition. However, a table with mean stride duration broken down per condition has now been added for additional information (Table 2).*

Line 284: “if stride length stays unchanged” – as far as I can tell, this was not quantified, so can you confirm that this is the case? Do you really expect that the horses trotted at a steady state and did not alter their stride length across these conditions? If not, how can you confidently infer that these horses actually trotted at a faster speed when interpreting your findings and clinical implications? I would be very cautious in making these statements, or at the very least, let the reader know that they should be cautious when interpreting your findings related to “trot speed”.

*Response: The reference to stride length has been removed. The slow/fast was assigned based on stride duration within each exercise condition and not in absolute terms (i.e. there was not cut off value for what slow or fast was between horses or condition.*

Line 286 and 323: what is meant by “on average horses altered their stride duration by 128 ms between the different exercise conditions”? this the average difference in stride duration between conditions? This is where a table for stride duration descriptive statistics would be helpful. What conditions can I expect to see this average increase in stride duration, and the resultant increase(?) in HDmin and PDMin? More information required – again, the table for stride duration data would be very useful.

*Response: This has now been revised. A lungeing vs straight-line comparison was added for HDmin.*

Lines 299 – 303: run on.

*Response: Edited.*

Line 305: why was this stride duration chosen?

*Response: The estimated marginal means are evaluated at mean stride duration (when stride duration is entered as covariate). However, this sentence has now been removed for brevity as only trends were identified in this case.*

Line 306: “limb to which the predominant asymmetry was attributed to on the inside” this is repetitively used throughout the manuscript and is such a lengthy description. Consider including this definition in the methods section and then using a shorter description like “affected/assigned (?) limb on inside or outside of circle”. It would make for better readability throughout.

*Response: ‘Assigned limb’ is not introduced in the methodology and used throughout the manuscript where relevant.*

Line 317: re-word “significant interaction revealed that PDMin was affected by..”

*Response: This has now been edited.*

Doesn’t line 321 repeat line 318?

*Response: This has now been edited.*

DISCUSSION:
The discussion section is far too long, which makes it very difficult to identify the main take-away messages from this work. Please make this section more concise and try to focus on significant findings instead of discussing every variable/condition.

*Response: Thank for your feedback and suggestions. The discussion section has now been reorganised and edited significantly to address the points below.*

Line 355 – 358: run-on. Please revise so that the introductory sentence is a clear, concise description of what you did.

Lines 358 – 364 do an excellent job of summarising the main findings. However, because you did not directly measure trot speed, I would suggest altering lines 364 – 366 to state that “changes in stride duration resulted in significant differences in X, Y, Z movement asymmetries, which suggests that handlers should endeavour to keep trot speed consistent during clinical evaluations”.

*Response: This has now been edited.*

Line 376: why might this be? Please use the discussion section to identify reasons for why your findings agree with, or differ from, other studies. Were there methodological reasons that might explain these differences between studies? Line 383, are you inferring that the movement asymmetries in your study are due to laterality and normal biological variation and this is why you didn’t see significant differences during lunging conditions? Can you be sure that this is the only reason, as horses were not clinically assessed for lameness? More detail needed and methodological limitations of this study should be considered when interpreting findings/comparing with other studies.

Line 380: “increases in head movement asymmetry were not identified”.

Line 388: “higher peace vertical forces increased load placed on affected structure”? please revise.

Line 394: “suggests”. How might they be affected differently by changes in stride duration/”speed”?

Line 396: please be sure to clearly state where results are drawn from previous studies, as it is sometimes hard to differentiate between discussion of your own findings and those of others. “Previous studies have demonstrated and effect of surface type on trot speed….”

Line 401: as “stride duration and speed” – please be clear that you only measured stride duration and are making inferences about speed from these data.

Line 407: repetitive, please remove.

*Response: Edited.*

Line 413: why is this?

Line 418/419: “decreasing vertical displacement of the pelvis” instead of “lower rise”. This line needs a reference.

Line 422: asymmetric hindlimb propulsion.

Lines 424 – 427: run on sentence and repeats results. Revise.

Lines 434 – 446: Caution should be taken when stating that surface type has no influence on pelvic movement asymmetries, especially given the limitations of this study where a variety of surfaces were used/not standardised, which will undoubtedly have influenced your findings. Please make this clear to the reader and avoid sweeping statements.

*Response: Population differences highlighted and the variation in surfaces is now highlighted in the limitations.*

Lines 436 – 440 – run on sentence, revise.

Doesn’t line 441 contradict your statements in the previous paragraph?

Line 449: “significant”.

Line 452: “moderate”.

Line 456: “influences”

*Response: All of the above now corrected.*

Line 455: The authors need to consider the influence of steady state treadmill vs. overground locomotion when making this type of claim. Please revise as it is a sweeping statement.

Line 466: So what can we draw from these findings in relation to stride duration and pelvic movement asymmetries? The take home message is unclear.

“Implications for Clinical Practice” – consider removing this section. It is highly repetitive (of results and previous parts of the discussion section) and will help to make your discussion section more concise. Further, and importantly, is it wise to extrapolate the results of this study into clinical practice? There are several methodological limitations that influence the validity of these findings and surely more standardised work is required before clinical recommendations can be made.

*Response: This section has now been removed.*

Line 472 “in clinical practice”.

Line 476: very large variation, hence my point above.

Lines 474 – 487: repeats what has already been presented in results and discussion. The question of “how tightly does speed need to be controlled in a clinical setting” is never answered. Can you make this conclusion based on your measurements in this study?

Line 490: Second to what? Again, this paragraph is repetitive and does not provide recommendations for clinical practice. Please remove.

Line 510 – 518 is well written and could be incorporated elsewhere in the discussion section.

Limitations: several of the main limitations of this study are not addressed in this section.

Line 524: these are mainly treadmill studies where velocity is standardised. The limitations related to extrapolating changes in stride duration with speed during overground locomotion need to be clearly highlighted to the reader, especially if you’re going to recommend that SD could be a proxy for speed (which I would recommend removing).
The lack of standardisation of surfaces, stride velocity etc need to be highlighted as limitations of this study as these are confounding factors that will undoubtedly have influenced your results. Further, my above concerns regarding the groupings/analysis of horses with inconsistent/vs consistent movement asymmetries should be addressed in this section.

Conclusion:
Line 548: “decreased”.

*Response: Edited*

Lines 547 – 552: extremely vague – does not leave the reader with a good take-away message relating to how movement asymmetries are affected by “circle direction”. Please look to the conclusions drawn in the abstract and try to incorporate these in the conclusions section to improve the take-away message.

*Response: This has now been edited*
